# The influence of rhythm on detection of auditory and vibrotactile asynchrony

**DOI:** 10.1007/s00221-019-05720-x

**Published:** 2020-03-04

**Authors:** Andrew P. Lauzon, Frank A. Russo, Laurence R. Harris

**Affiliations:** 1 Department of Psychology, York University, 4700 Keele St, Toronto, ON M3J 1P3 Canada; 2 Department of Psychology, Ryerson University, Toronto, ON Canada; 3 Centre for Vision Research, York University, Toronto, ON Canada

**Keywords:** Simultaneity perception, Entrainment, Rhythm, Temporal asynchrony detection thresholds, Dynamic attending

## Abstract

The perception of an event is strongly influenced by the context in which it occurs. Here, we examined the effect of a rhythmic context on detection of asynchrony in both the auditory and vibrotactile modalities. Using the method of constant stimuli and a two-alternative forced choice (2AFC), participants were presented with pairs of pure tones played either simultaneously or with various levels of stimulus onset asynchrony (SOA). Target stimuli in both modalities were nested within either: (i) a regularly occurring, predictable rhythm (ii) an irregular, unpredictable rhythm, or (iii) no rhythm at all. Vibrotactile asynchrony detection had higher thresholds and showed greater variability than auditory asynchrony detection in general. Asynchrony detection thresholds for auditory targets but not vibrotactile targets were significantly reduced when the target stimulus was embedded in a regular rhythm as compared to no rhythm. Embedding within an irregular rhythm produced no such improvement. The observed modality asymmetries are interpreted with regard to the superior temporal resolution of the auditory system and specialized brain circuitry supporting auditory–motor coupling.

**Electronic supplementary material:**

The online version of this article (10.1007/s00221-019-05720-x) contains supplementary material, which is available to authorized users.

## Introduction

Rhythm is a fundamental and ubiquitous context capable of providing a predictive framework that can inform judgments about the temporal sequencing of events. The presence of a regularly occurring rhythm has been shown to boost performance on auditory psychophysical tasks such as judged duration of time intervals (Barnes and Jones 2000; Jones and Yee 1997; Large and Jones 1999), detection of tones in noise (ten Oever et al. 2014), and judgements of pitch, with participants showing greater accuracy and decreased reaction times when targets occur on the expected beats of a regular rhythm as opposed to within the context of an irregular rhythm or with no rhythm at all (Ellis and Jones 2010; Jones et al. 2002). Here, we explore the effect of rhythm on asynchrony detection in auditory as well as vibrotactile domains.

Humans routinely synchronize their movements to regular rhythms, producing a form of temporal coupling commonly referred to as sensorimotor synchronization (London 2012), which often manifests as foot or finger tapping. Sensorimotor synchronization is thought to depend on neural entrainment to the beat (Repp 2005), which involves activation of brain areas commonly associated with motor planning such as the pre-motor cortex, supplementary motor area, basal ganglia, and cerebellum (Chen et al. 2008; Grahn and Rowe 2009). This activation occurs under passive listening conditions, which rules out the possibility that the motor activation is merely a consequence of movement. Furthermore, ffresearch using electroencephalography (EEG) has demonstrated that steady-state-evoked potentials robustly couple with regular rhythms (Nozaradan et al. 2012). Schroeder and Lakatos (2009) have proposed that periodic neural oscillations reflecting alternating phases of high and low neural excitability might influence processing of sensory input. In particular, the processing of stimuli that coincide with peaks in neural excitation may be facilitated. Jones et al. (2002) foreshadowed these neurally based proposals with a complementary theory referred to as dynamic attending, in which periodic pulses of heightened attention entrain to predictable events such as a regular rhythm. Both of these theories offer a possible explanation for the aforementioned rhythmically enhanced detection and discrimination results, and prompt the question of whether other psychophysical tasks such as detection of asynchronous events may also benefit from entrainment.

Asynchrony detection has been extensively studied in the auditory domain, and thresholds are commonly reported as being under 10 ms (Babkoff 1975; Corso 1978; Fraisse 1963*)* and even as low as 2 ms (Exner 1875); however, we are not aware of any studies investigating the influence of rhythmic context on asynchrony detection. We also wondered whether rhythmic context might have an influence in asynchrony detection for non-auditory stimuli. Wing and Kristofferson (1973) proposed an internal timekeeper model that is amodal and mediated by higher cognitive processes. While this model has been successful in explaining a variety of temporal phenomena, there is also a considerable body of evidence supporting the concept of an “auditory advantage”, i.e., that audition is superior to all other modalities for any temporal processing task (Repp and Penel 2004; Iversen et al. 2015; Ammirante et al. 2016). These results imply a modality weighting, whether mediated by differences in early-stage or late-stage perceptual processes.

There is an inherent relationship between sound and vibration, and numerous parallels in the ways humans process and perceive sound and touch stimuli (von Bekesy 1959). Music listeners speak of “feeling the beat” and there is often a tactile component to rhythm perception, a point drummers and dancers can attest to. Asynchrony detection tasks have been carried out with somatosensory input, using a variety of stimuli including vibrotactile stimulation (Petrosino and Fucci 1984), mechanical taps (Gescheider 1966), and electrocutaneous shock (Uttal 1959; Rosner 1961). The locus of stimulation has varied considerably across studies, with common sites being fingertips (Elliott et al. 2010; Huang et al. 2012), tongue, and thenar eminence (Petrosino and Fucci 1984). Substantial variability in asynchrony detection thresholds has been found in tactile studies, possibly attributable to the variation in stimuli type and the location of stimulation. Nevertheless, thresholds tend to be in the range of 10–30 ms, which is higher than those typically obtained in auditory studies (Petrosino and Fucci 1984, 1989; Rosner 1961; Uttal 1959), and have been observed to be as high as 179 ms (Laasonen and Virsu 2001). Von Bekesy (1959) found that vibrotactile sensations required 5–10× the amount of stimulation time to be perceived at their full magnitude as compared to hearing sensations. Regardless of absolute values, the auditory advantage in asynchrony detection has been further supported by studies that have made direct comparisons between auditory and somatosensory inputs (Gescheider 1966; Hirsh and Sherrick 1961; Laasonen and Virsu 2001).

The present study was conducted to: (a) test the hypothesis that the threshold at which participants were capable of detecting asynchrony between two events would be decreased when placed in a rhythmic context; and (b) to assess whether this rhythmic advantage manifests in somatosensory as well as auditory modalities.

## Methods

### Participants

Ten volunteers (four females, six males, age range 22–48 years; mean age = 31.7 years) were recruited by word-of-mouth. They all reported normal hearing. Participants received no financial compensation.

### Apparatus

**Fig. 1:**
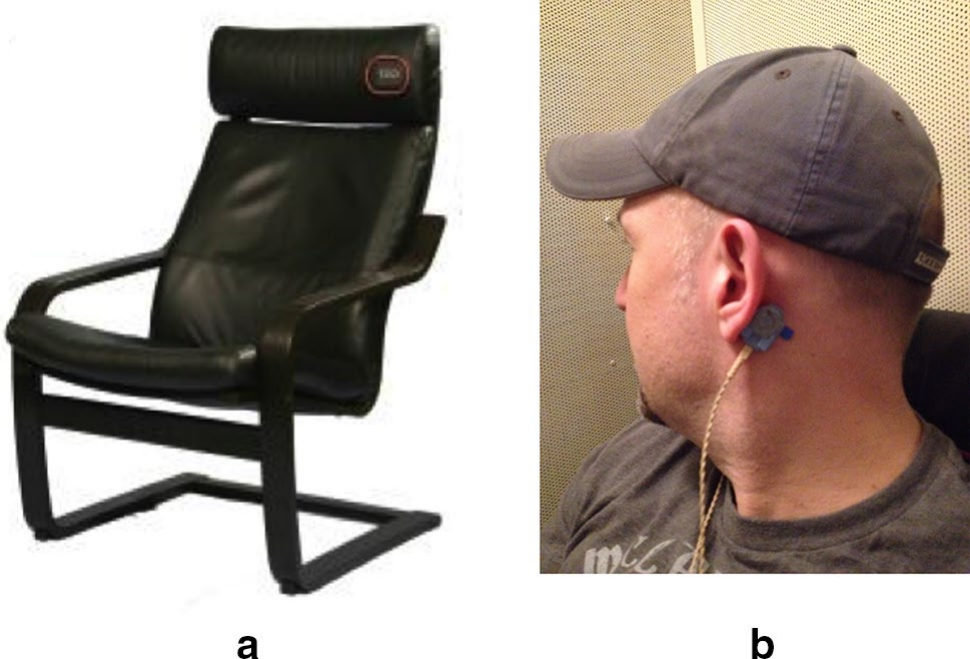
**a** The Emoti-Chair and **b** transducers were attached to the mastoid bones. They emitted pink noise during each trial to mask bone conduction of chair vibrations

Stimuli were either sounds played through headphones or vibrotactile stimulation applied to the lower back. Stimulus presentation was controlled using Cycling’74 Max MSP software running on a 2010 Macbook Pro with a 2.66 GHz Intel Core i7 processor and 4 GB of DDR3 RAM. An RME Fireface 400 FireWire audio interface was used to direct six channels of audio output from the Macbook. Two channels of audio output were directed to a Behringer MX602a analog mixing console and delivered to participants via Sennheiser HD518 over-ear headphones. Each ear received the same signal. Four additional channels of audio output were directed to four voice coils (each 1″ in diameter) embedded in the seat and back of a padded form-fitting chair (Fig. 1a, Emoti-Chair; Karam et al. 2009). Pink noise was delivered in each trial through the headphones to mask any air-conducted sound originating from the voice coils. In addition, pink noise was delivered through Tactaid VBW32 bone-conduction transducers placed on the left and right mastoids. This latter procedure was adopted to mask any residual sound originating from the voice coils (Fig. 1b; after Russo et al. 2012). This setup for vibrotactile stimuli and masking was modeled after the conditions used by Ammirante et al. (2016) that led to equivalent sensorimotor synchronization across vibrotactile and auditory rhythms. Notably, this prior study found modality equivalence under conditions where the rhythm was metronomic and the area of vibrotactile stimulation was relatively large, spanning the buttocks (2 channels) and lower back (2 channels).

Prior to experimental trials, stimulus levels were adjusted to equalize the perceived magnitude of the auditory and vibrotactile stimuli: all three authors corroborated the levels. Participants were asked if they could hear the chair vibrations during the experiment and all reported that they could not. The sound level of stimuli 12″ (30.5 cm) from the chair surface was approximately 90 dB SPL, as measured using a B&K 2250 sound-level meter with a B&K ZC-0032 pre-amp and a pre-polarized free-field ½” type 4950 microphone.

### Stimuli

**Fig. 2:**
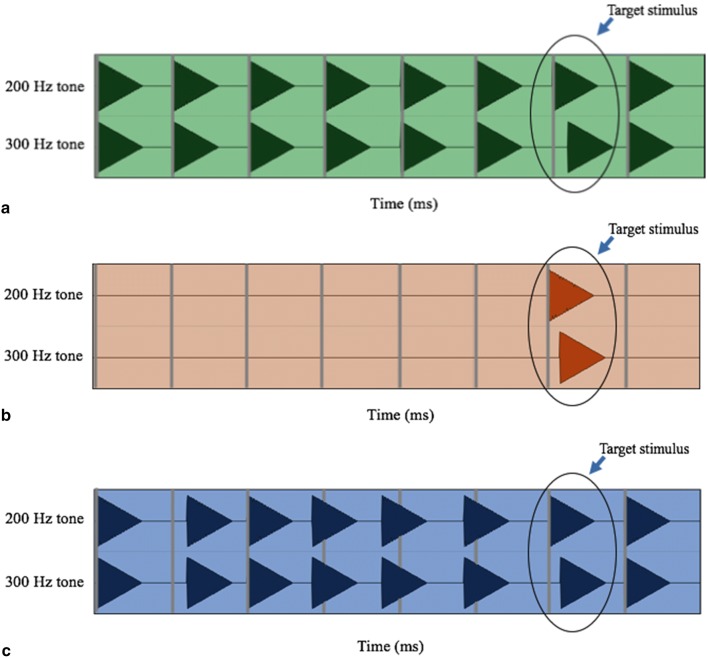
Rhythmic context conditions. Each triangle represents the amplitude envelope of each tone. Both components of the stimuli were presented through the same loudspeakers (in the headphones or chair). Vertical grey bar lines show increments of 500 ms. **a** Regular-rhythm condition. **b** No-rhythm condition. **c** Irregular-rhythm condition. Inter-stimulus intervals varied from 400 to 665 ms in an unpredictable pattern (Images are screen captures from Avid Pro Tools, annotated using Microsoft Powerpoint.)

Each target and context stimulus consisted of a pair of sinusoidal vibrations (200 and 300 Hz), presented through headphones in the auditory conditions, and via voice coils embedded in the Emoti chair in the vibrotactile conditions. Target stimuli were presented in one of the three different rhythmic contexts:

*Regular rhythm* (RR; Fig. 2a). Eight beats of 200/300 Hz pure tone pairs were played with an inter-beat interval of 500 ms (120 beats per minute, BPM). Beats 1–6 and 8 were the context stimuli, with the tones played in perfect synchrony. Beat 7 was the target stimulus played with either one of the ten pre-selected delays SOA or in synchrony.

*No rhythm* (NR; Fig. 2b). Target stimuli were presented with no context. This condition had the identical timing as the rhythm conditions, except that there were no context stimuli (beats 1–6 and 8).

*Irregular rhythm* (IR; Fig. 2c). Target stimuli were presented within the context of an irregularly occurring, unpredictable beat sequence. This was identical to the regular-rhythm condition except that the beat interval duration varied pseudo-randomly between 400 and 667 ms (90–150 BPM) on each of the first six beats. Target stimuli occurred at the same time within each trial as they did in both the other conditions.

In each trial, the target stimulus consisted of a pair of pure tones presented at 200 and 300 Hz. The resulting frequency ratio (2:3) is considered in Western harmony to be the most consonant interval after the unison (1:1) and octave (1:2). We avoided the unison because of potential amplitude variations resulting from phase differences between tones, and we avoided the octave as it has previously been shown to cause confusion in auditory temporal discrimination tasks (Hirsh 1959).

Each tone had an instantaneous attack and a 300 ms linear decay (see Fig. 2). The two tones were presented either in perfect synchrony or with one of ten stimulus onset asynchronies (SOA). SOA pilot trials confirmed that sensitivity to asynchrony for auditory and vibrotactile stimuli were in different ranges, and so, it was not possible to use the same range of SOAs for both modalities. Ranges for each modality were chosen by running the experimenters through pilot trials and adjusting the ranges so as to leave enough room at either end to avoid possible ceiling or floor effects. The SOA range was set at 5–23 ms in increments of 2 ms for auditory stimuli, and at 10–190 ms in increments of 10 ms for vibrotactile stimuli. Each modality had a total of ten discreet SOA values. Example trials and the Max MSP scripts used to run the experiment are available under “Online Resources”.

### Procedure

Participants sat in the Emoti-Chair wearing the headphones and Tactaid mastoid stimulators. For each trial, and for each condition, participants were exposed to the context rhythms and target stimulus sequence twice: once with the asynchrony at beat seven and once with no asynchrony. Each of the ten SOA values was presented ten times, for a total of 100 trials per condition. The order of presentation was randomized via the Max MSP script and the stimulus trains were separated by a random interval ranging from 2 to 4 s. Pink noise commenced 1 s prior to the first stimulus presentation and continued until the end of the second-stimulus presentation. Participants indicated in which sequence (first or second) the asynchrony occurred by entering either “1” (for first) or “2” (for second) on a computer keyboard. This 2AFC method was chosen to avoid response bias. The next trial began once a response was entered. A block design was employed with each block consisting of either auditory or vibrotactile stimulation with one of the three rhythmic contexts. The order was counterbalanced between subjects.

Participants were given an orientation session prior to the experimental trials in which they were familiarized with the sound and feel of each pure tone played separately, synchronously, and asynchronously. They were also given approximately ten practice trials prior to commencing each block until they reported feeling confident in the task.

### Data analysis

A percent correct score was calculated for each SOA, and a logistic curve between 50% (chance) and 100% correct was fitted to each participant’s datum for each of the six conditions using Eq. 1. Curves were fit using Sigmaplot, which uses a Marquardt–Levenberg algorithm.

1 $$ {\text{Percent correct }} = \, 0.50 + 0.50/\left( {1 + {\exp}\left( { - \left( {x - x_{0} } \right)/b} \right)} \right), $$

where *x*_0_ is the 75% threshold and *b* is the standard deviation which we take as our measure of variability.

Data analyses were then conducted on the 75% threshold and standard deviation values.

Separate repeated-measures ANOVAs were performed within each modality to compare thresholds, and two-way repeated-measures ANOVAs were performed to compare overall mean thresholds and standard deviations between the two modalities. Pairwise comparisons used Bonferroni correction.

## Results

### Detection thresholds

**Fig. 3:**
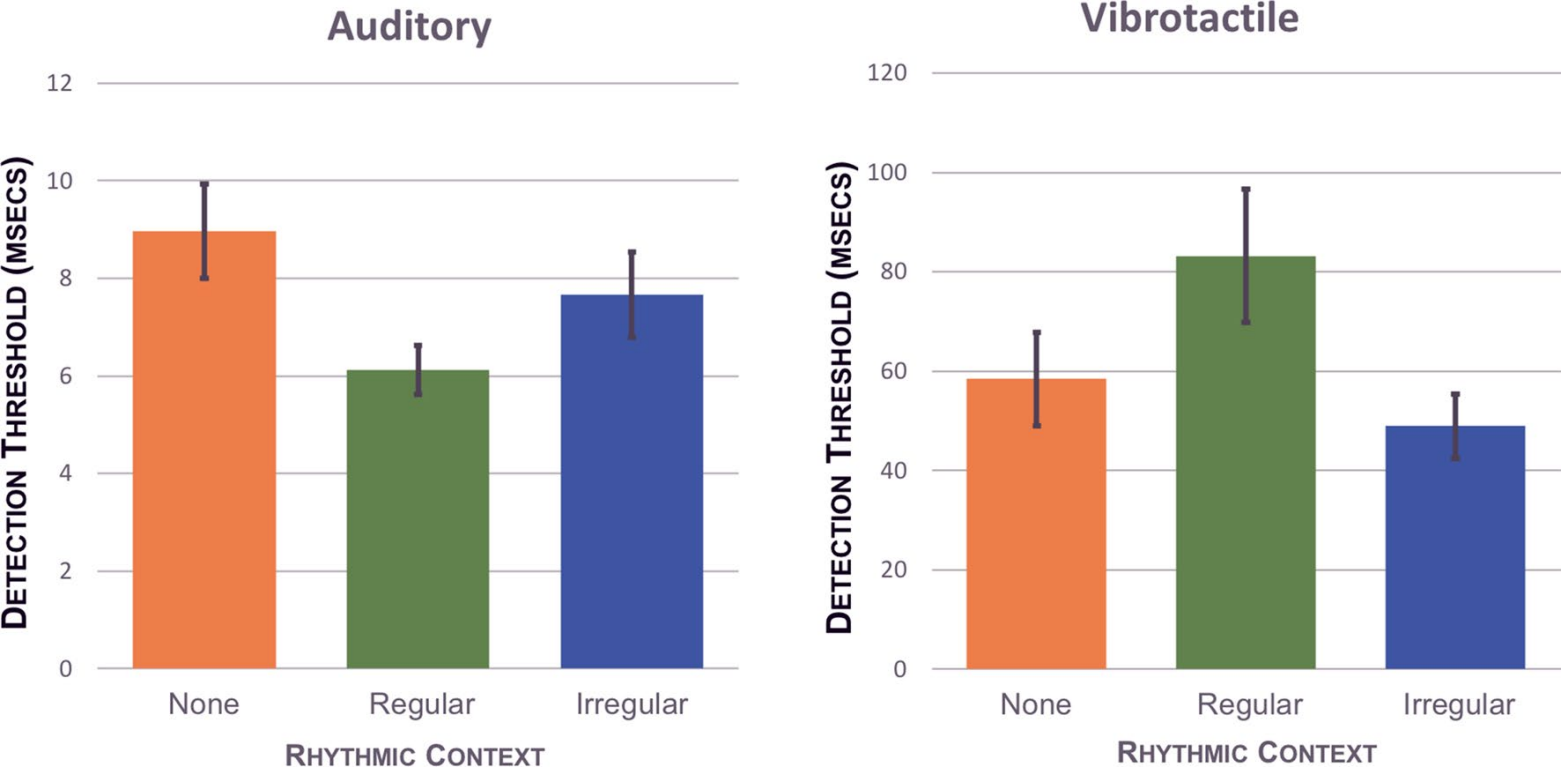
Mean thresholds of detection compared within modalities. Error bars represent standard error of the mean. Left: detection thresholds for the auditory conditions. Right: detection thresholds for vibrotactile conditions. Note the difference in vertical scales. Asterisk indicates *p* < 0.05 (Images created using Microsoft Excel.)

Figure 3 shows the average detection thresholds for the three rhythmic contexts within each modality. A preliminary ANOVA indicated that detection thresholds were greater for the vibrotactile condition than the auditory condition, *F*(1, 9) = 92.31, *p* < 0.001, partial *η*^2^ = 0.911. One-way repeated-measures ANOVAs performed within each modality revealed a significant effect of rhythmic context in the auditory condition, *F*(2, 18) = 3.56, *p* = 0.05, partial *η*^2^ = 0.283, but no significant effect of rhythmic context in the vibrotactile condition, *F*(2, 18) = 2.86, *p* = 0.08, partial *η*^2^ = 0.241. Pairwise comparisons revealed that detection thresholds in the auditory no-rhythm condition (*M* = 8.97 ms, SD = 3.06 ms) were significantly higher than the regular-rhythm condition (*M* = 6.12 ms, SD = 1.60 ms), (MD = 2.85 ms, SE_m_ = 0.93, *p* = 0.04); however, there was no significant difference between the regular-rhythm condition and the irregular condition (MD = 1.54 ms, SE_m_ = 0.96, *p* = 0.43).

### Standard deviations

**Fig. 4:**
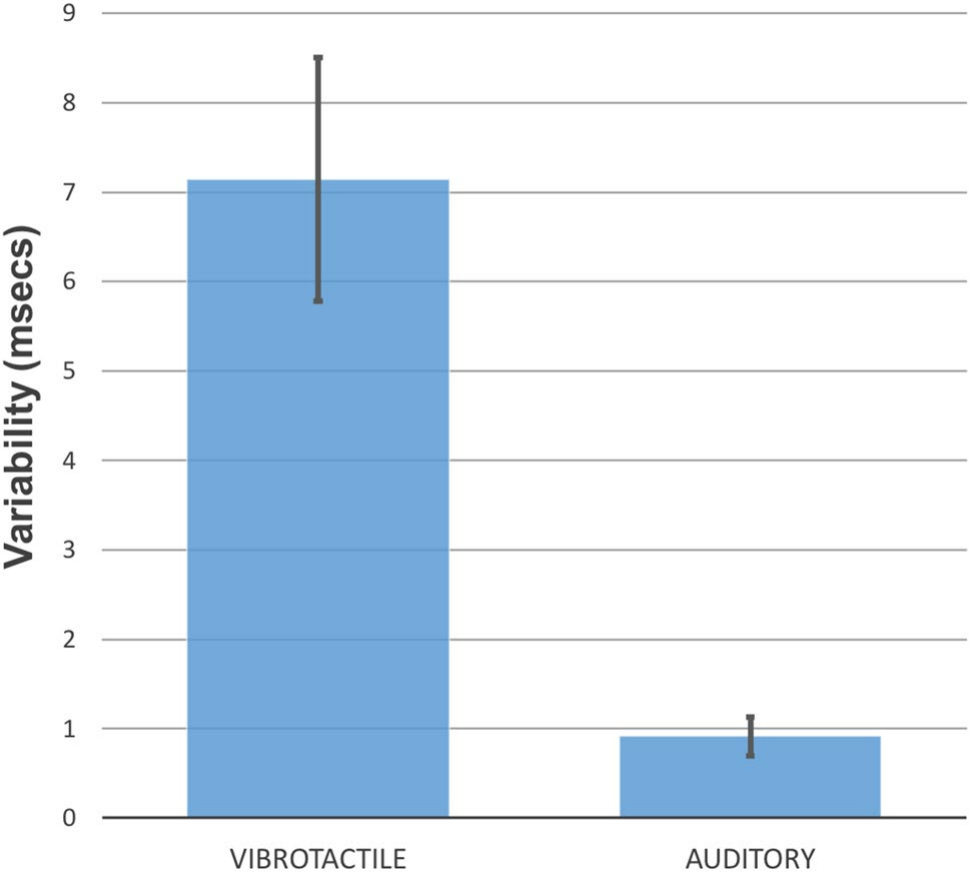
Comparison of overall variability of responses in each modality. Error bars represent standard error of the mean (Image created using Microsoft Excel.)

Figure 4 plots the average standard deviations of the detection thresholds for the rhythmic contexts for each modality. A 2 (modality) × 3 (rhythmic context) repeated-measures ANOVA was performed. The results show a significant main effect of modality, *F*(1, 9) = 23.51, *p* = 0.001, partial *η*^2^ =0.723, indicating that variability differed between modalities. The vibrotactile condition had higher levels of variability compared to the auditory condition (MD = 6.23 ms, SE = 1.28, *p* = 0.001). No significant differences in response variability were found between rhythmic contexts of either modality.

## Discussion

In the present study, participants showed greater sensitivity to auditory asynchrony detection when stimuli were embedded within a regular rhythm as compared to stimuli presented with no rhythmic context (~ 32% improvement). Embedding in an irregular rhythm did not produce any such improvement. Although there was no significant difference found between asynchrony detection when stimuli were embedded within a regular rhythm compared to an irregular rhythm, the mean threshold for the irregular rhythm was numerically in-between the regular-rhythm and no-rhythm conditions. The lack of a significant difference between regular and irregular rhythm was a somewhat surprising result, considering previous research, showing that regular rhythms can facilitate robust enhancements of reaction times and stimulus detection accuracy over irregular rhythms (Morillon et al. 2016; Rimmele et al. 2011; Rohenkohl and Nobre 2011; Rohenkohl et al. 2012). One possible explanation for the absence of an effect in this case may be the small sample size of the present study.

Vibrotactile asynchrony detection was much less sensitive than auditory asynchrony detection with mean vibrotactile tactile thresholds of 63.5 ms compared to mean auditory thresholds of 7.6 ms and showed much higher variability. Furthermore, these thresholds showed no improvement when the target stimulus was embedded in a regular rhythm. Although not significant, the regular rhythm actually led to numerically worse performance.

### The effect of rhythm on auditory asynchrony detection: attention and neural entrainment

One possible explanation for the reduced asynchrony detection thresholds obtained in the auditory rhythm condition is that the rhythm served as a predictive framework informing the temporal sequencing of events. If a participant could anticipate the time of arrival of each beat of the stimulus, then he or she would have a small window of time in which to focus attention, an idea described by dynamic attending theory (Jones et al. 2002). Dynamic attending theory suggests that stimulus-driven attentional pulses are entrained to a regular pattern, such as the pattern used in our study. By this account, attention would be maximally focused on each beat (every 500 ms in this case), and thus, participants would be less susceptible to errors borne of inattentiveness. The same theory has also been used to explain rhythmically enhanced performance on other, similar psychophysical tasks in the auditory domain (Barnes and Jones 2000; Ellis and Jones 2010; Jones et al. 2002; Jones and Yee^1997^; Large and Jones 1999; ten Oever et al. 2014).

It has been suggested that attention can operate in a “rhythmic mode” when neural oscillations entrain to exogenous stimuli, enhancing sensory input and anticipatory responses during the periods of heightened neuronal excitability that accompany each beat (Rohenkohl and Nobre 2011; Schroeder and Lakatos 2009). If these periodic neural oscillations also facilitated temporal expectation and attentional dynamics, and both processes contributed to enhanced perception, the reduced detection threshold effect observed in this experiment would be a result of a complex dynamical system.

But why was the same rhythmic effect not present in the vibrotactile modality? Much like the auditory cortex, the somatosensory cortex has strong connectivity with motor areas involving feedforward and feedback pathways (Christensen et al. 2007 ). The somatosensory feedback pathways are thought to be particularly important in underpinning the adaptive precision grip, allowing for secure handling of objects between the fingertips across a range of conditions (Witney et al. 2004). However, there may be something fundamentally different about the auditory feedback pathways. In particular, these feedback pathways may be modulated by oscillatory subcortical activity, especially in the basal ganglia, that has entrained to an external rhythm (Grahn and Brett 2007; Grahn and Rowe 2009). It has been suggested that this capacity for auditory rhythmic entrainment may have arisen to support vocal learning (Patel and Iverson 2014). In support of this view, flexible synchronization with rhythm has been observed in vocal-learning species that are only distally related to humans (e.g., parrots and elephants), but has not been observed in non-human primates (Merchant et al. 2015). Although several studies have elicited what appears to be entrainment to vibrotactile rhythms under certain conditions (e.g., Ammirante et al. 2016; Brochard et al. 2008; Elliot et al. 2010; Huang et al. 2012), it is quite possible that this capacity is mediated by auditory–motor connectivity.

Another possible account for the rhythmic simultaneity advantage observed in the auditory condition involves modality differences in working memory. While the 2AFC method may be effective in controlling for response bias, the participant is required to retain a memory trace of the first-target stimulus for comparison to the second, which happens a few seconds later, and in some cases, following the presentation of context stimuli. It seems likely that it is more difficult to retain a memory trace for a tactile stimulus compared to an auditory stimulus (Bancroft and Servos 2011; Bancroft et al. 2011; Gallace et al. 2008; Harris et al. 2001). Even in the absence of formal musical training, all participants would have accumulated substantial experience with auditory working memory in the context of music listening. Future studies might consider alternate experimental designs that reduce the memory component.

An alternative and somewhat more parsimonious explanation for the rhythmic simultaneity advantage observed in the auditory condition may be found in differences in temporal precision between the modalities. The somatosensory system does not appear to be capable of the temporal precision afforded by the auditory system, as evidenced by the large thresholds and high variability in simultaneity judgements observed, regardless of rhythm condition. Thus, while vibrotactile stimuli may be capable of generating rhythmic entrainment in some contexts (Ammirante et al. 2016; Brochard et al. 2008; Elliott et al. 2010; Huang et al. 2012), any potential benefit of this entrainment is likely insufficient to overcome the poor temporal resolution of the somatosensory system as a whole.

## Conclusion

This study showed that context in the form of a regular-rhythmic pattern improves the capacity to detect asynchronous events in the auditory domain. The high variability of response and the absence of significant effects of rhythmic context in the vibrotactile modality suggest a greater tolerance in the somatosensory system for interpreting stimuli as synchronous. Our results also lend further support to the privileged status of the auditory system for temporal processing and sensorimotor synchronization.

## Electronic supplementary material

Below is the link to the electronic supplementary material.

Irregular Rhythm Asynchronous: Example auditory trial of the asynchronous irregular rhythm condition. Masking noise not included (WAV 778 kb)

Irregular Rhythm Synchronous: Example auditory trial of the synchronous irregular rhythm condition. Masking noise not included (WAV 776 kb)

No Rhythm Asynchronous: Example auditory trial of the asynchronous no rhythm condition. Masking noise not included (WAV 228 kb)

No Rhythm Synchronous: Example auditory trial of the synchronous no rhythm condition. Masking noise not included (WAV 170 kb)

Regular Rhythm Asynchronous: Example auditory trial of the asynchronous regular rhythm condition. Masking noise not included (WAV 730 kb)

Regular Rhythm Synchronous: Example auditory trial of the synchronous Regular rhythm condition. Masking noise not included (WAV 740 kb)

Max MSP Patches: Source files for the script used to run the experiment. Each condition utilizes a different patch (included). Cycling ‘74 Max MSP software is required to run the scripts (MAXPAT 171 kb)

Max MSP Script - Audio Random (MAXPAT 637 kb)

Max MSP Script - Audio Regular (MAXPAT 159 kb)

Max MSP Script - VT Irregular (MAXPAT 169 kb)

Max MSP Script - VT Random (MAXPAT 138 kb)

Max MSP Script - VT Regular (MAXPAT 134 kb)
